# Cross-Regulation between Transposable Elements and Host DNA Replication

**DOI:** 10.3390/v9030057

**Published:** 2017-03-21

**Authors:** Mikel Zaratiegui

**Affiliations:** Department of Molecular Biology and Biochemistry, Rutgers, the State University of New Jersey, 604 Allison Rd, Nelson Biolabs A133, Piscataway, NJ 08854, USA; zaratiegui@dls.rutgers.edu; Tel.: +1-848-445-1497

**Keywords:** transposable elements, DNA replication, replication fork, transcription, genome integrity

## Abstract

Transposable elements subvert host cellular functions to ensure their survival. Their interaction with the host DNA replication machinery indicates that selective pressures lead them to develop ancestral and convergent evolutionary adaptations aimed at conserved features of this fundamental process. These interactions can shape the co-evolution of the transposons and their hosts.

## 1. Introduction

Transposable elements (TE) are ubiquitous in the tree of life. They have colonized almost all genomes sequenced to date, throughout eukaryotic, prokaryotic and archaeal domains. TE maintain their presence in the host genome by increasing their copy number via transposition, and colonize new genomes through horizontal transfer. Through these activities, TE exert a major influence in the evolution of the species.

Like viruses, TE are molecular parasitic elements that contain few genes, and they must condense multiple activities to subvert cellular functions to enable their continued presence in the host genome. This paucity of genetic payload leads molecular parasites to focus their intervention towards very fundamental cellular processes. As a consequence, research into viruses has led to some of the most seminal discoveries in molecular biology, such as the mechanisms of eukaryotic DNA replication, mRNA processing and many others. Similarly, the investigation of the transcriptional and post-transcriptional regulation of eukaryotic TE has been very fruitful, advancing our understanding of transcription and chromatin dynamics [1,2].

The equally fundamental process of DNA replication is another point of interaction between parasites and their hosts. The potential of TE to influence host genome stability and evolution make this problem a subject of particular interest, because it could have direct implications in the etiology of diseases like cancer and aging. Since the influence of host DNA replication extends across both type I retrotransposons and type II DNA transposons, it is worthwhile to discuss them together. The purpose of this review is to summarize the current evidence of TE influence on host DNA replication and vice versa, and to speculate on the potential selection pressures that shape its evolution.

## 2. DNA Transposon Duplication

**Figure 1:**
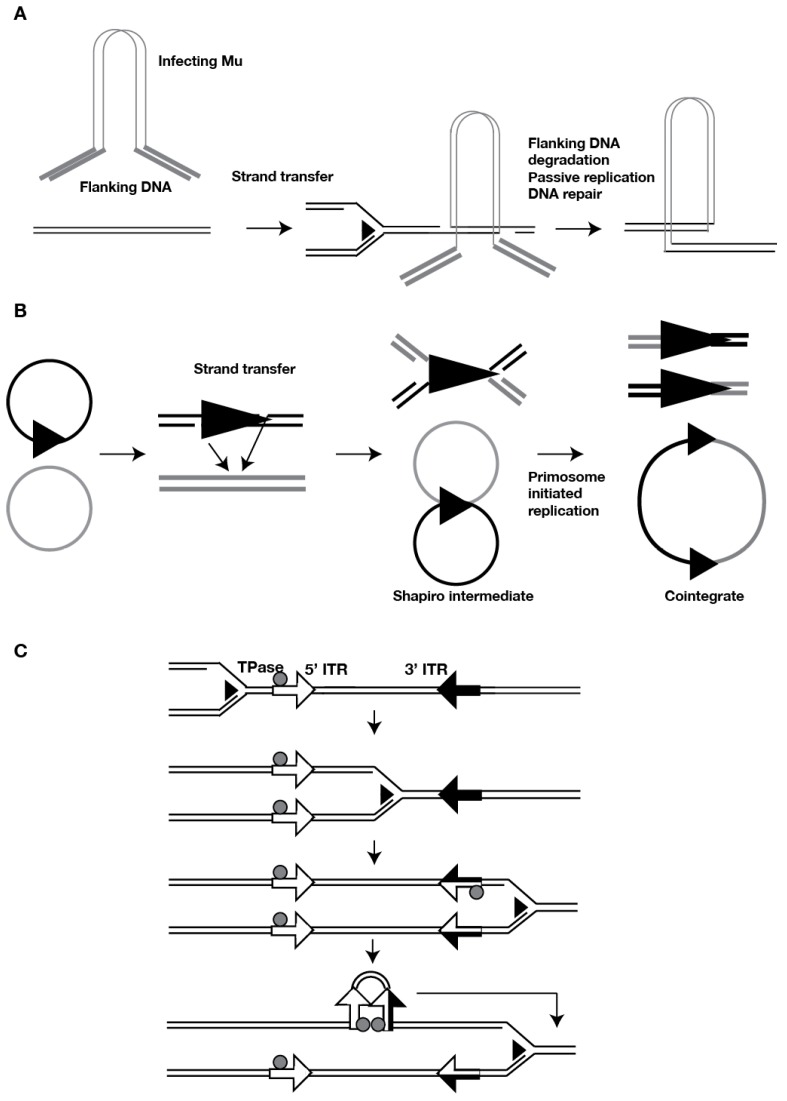
Replication of type II DNA transposons. (**A**) Non-replicative transposition of Mu after infection. The Mu phage and flanking DNA are injected into the host. Cleavage and strand transfer join the Mu phage DNA to the target site, leaving single-stranded gaps. Upon arrival of a replication fork the flanking DNA is degraded, and the gaps create a double stranded end create a double stranded end. Both gaps are simultaneously filled by passive DNA replication, yielding a mature prophage; (**B**) Replicative transposition of Mu in the lytic phase. Strand transfer of the prophage into the target site create a Θ-shaped Shapiro intermediate, with the Mu element flanked by fork-like structures. Primosome-started replication at these structures duplicate the Mu element in a joined cointegrate; (**C**) Control of activator/dissociator (Ac/Ds) transposition by replication fork passage. Methylation at the inverted terminal repeats (ITRs) is depicted as filled arrows. Hemimethylated ITR depicted as half-filled arrows, with the filled portion indicating the methylated strand. Replication of the methylated 3′ ITR yields two hemimethylated daughter ITR, only one of which binds the transposase (TPase), determining which of the two daughter elements can assemble the transpososome.

Type II elements, also known as DNA transposons, do not generate an RNA transposition intermediate, and they must rely on the host DNA replication machinery to increase their copy number. One way to do this is through a partial transposition in which a single strand of the donor copy is inserted in a target site, leaving DNA replication to generate the complementary strand of both the donor copy and the new insertion. The Mu phage and the bacterial Tn3 family of transposons can undergo such a replicative transposition through single-stranded donor cleavage and strand transfer into the target site, yielding a θ structure known as the Shapiro intermediate [3] (Figure 1). Subsequent DNA replication duplicates the joint insertion into a co-integrate, doubling the copy number. Similarly, the concerted model of Helitron transposition starts with single-stranded cleavage and 5′ strand transfer, followed by strand displacement of the transposed strand by replication from the free 3′ OH of the donor [4]. The displaced strand is cleaved and joined with the 5′ end of the target nick, leaving it as a heteroduplex that resolves by passive DNA replication, generating a new copy of the Helitron in one of the daughter strands.

Both of these strategies rely on DNA replication for completion of the transposition. The initial integration of infecting Mu (lysogenic stage) is a non-replicative transposition but it nevertheless depends on passive DNA replication for completion. In this case a native host-initiated DNA replication fork that encounters the transpososome directs the degradation of flanking DNA that usually accompanies the injected Mu phage DNA, and repairs the gaps resulting from the staggered nicks in the insertion site, resulting in a mature prophage [5] (Figure 1A). In contrast, the Mu phage in its lytic stage completes each new replicative transposition by the assembly of a primosome dependent “restart” replisome at the fork structures created by strand transfer [6] (Figure 1B). The switch from transposition to replication is coordinated by the transpososome in collaboration with host factors, and in this case, it can be said that the transposon machinery initiates replication.

The more prevalent canonical “cut-and-paste” transposition mechanism can also take advantage of passive DNA replication to increase copy number, by directing mobilization of a copy from one of the daughter chromatids generated after passage of the replication fork into an unreplicated region of the host genome. The transposition machinery can sense the replicated/unreplicated status of an insertion by measuring the level of DNA methylation of the two DNA strands. Nascent DNA is unmethylated, and DNA replication leaves transiently hemimethylated sites that are subsequently restored to full methylation by the maintenance DNA methyltransferases. A methylation-sensing mechanism has been demonstrated in multiple bacterial transposons and in the maize transposons activator/dissociator (Ac/Ds) (Figure 1C).

Ac/Ds transposes during DNA replication. Only one of the two daughter elements becomes active, and can transpose ahead of the replication fork to create a new insertion [7,8,9]. The cause of both the S-phase activity and the “chromatid selectivity” of mobilization was traced to the methylation status of the transposase (TPase) binding sites in the inverted terminal repeats (ITRs) [10]. The Ac TPase binds with strongly differential affinity depending on which strand is hemimethylated [11]. Since only the 3′ ITR presents high levels of methylation [12], passage of the fork leaves one daughter element that allows TPase binding at both ends because its 3′ ITR shows permissive hemimethylation. This element can actively mobilize, but the other daughter element, with non-permissive hemimethylation in the 3′ ITR, remains inactive [10].

Replication fork passage controls Tn10/IS10 transposition and chromatid selectivity by a very similar mechanism, utilizing the Dam methylation motif to control binding of the TPase. In addition, hemimethylation of the Dam motifs allow binding of the RNA polymerase and transcription of the TPase gene, further coupling transposition to DNA replication [13].

Type II transposons are much less prevalent in mammals, and a potential role of replication fork passage sensing is yet to be demonstrated. The human Tc1/mariner family element HsMar1 represents a potential example. HsMar1 transpososome formation is sensitive to DNA topology, and is enhanced by negatively supercoiled DNA that could occur in the wake of the replication fork [14,15]. Besides DNA methylation, other epigenetic marks that exhibit slow re-establishment in the wake of the fork, such as Histone 4 Lysine 20 methylation [16], may also regulate transposon activity in eukaryotes [17].

## 3. Role of the Replication Fork in Transposon Target-Site Selection

The involvement of replication forks extends into the insertion stage of mobilization. The functional implications are difficult to gauge because they often involve essential cellular functions. Nevertheless, evidence from TE representing multiple classes of elements, both type I and type II, point to a direct role of replication fork dynamics in target site selection and the nucleic acid transactions that underlie insertion.

One point of cross-talk between transposons and the replication fork that extends across type I and type II elements in eukaryotes and prokaryotes is the interaction between the transposition machinery and the sliding clamps that coordinate replisome function. This activity is a universal requirement for processive DNA replication, and is carried out by proteins showing the DNA clamp fold, which forms multimers that encircle double stranded DNA [18]. The bacterial sliding clamp is a homodimer of the beta subunit of DNA polymerase III (Pol III; β-clamp), while in Archaea and Eukaryotes it is formed by a homotrimer of the proliferating cell nuclear antigen (PCNA).

Sliding clamps recruit a myriad of proteins involved in DNA replication, DNA repair and, in the case of PCNA, chromatin assembly. The majority of these interactors bind via hydrophobic pockets on the advancing face of the sliding clamp, gaining access to the primer terminus of the nascent DNA. These conserved hydrophobic domains recruit proteins sporting consensus binding motifs: β-clamp interactors show QxxL(x)F or QL(S/D)LF, and PCNA interactors show a remarkably similar sequence known as PCNA Interacting Protein motif (PIP-box: Qxx[I/L/M]xxF[F/Y]). The first transposon protein observed to interact with a sliding clamp was the *Drosophila melanogaster* type II POGO TPase, which was identified as a PCNA interactor in a yeast two-hybrid screen [19]. It exhibits a PIP-box that is conserved in its human relative, Tigger, and in the pogo-like Arabidopsis element Lemi1 [20]. A putative PIP-box can also be observed in the maize Ac/Ds transposon. However, the functional relevance of these motifs remains unclear.

**Figure 2:**
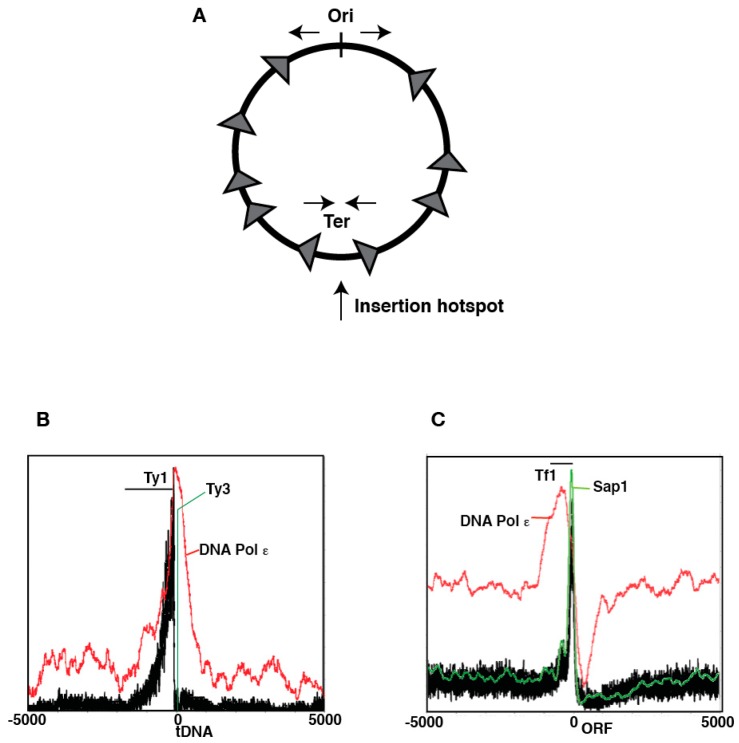
Fork influence on target site selection. (**A**) Insertion patterns of the Tn7 TnsE-dependent transposition into the host chromosome. Ori = origin of replication. Ter = replication termination region. Insertions are depicted as grey arrows; (**B**) Insertion patterns of Ty1 and Ty3 in type III genes. Ty1 insertions in black [33], Ty3 insertions in green [34] DNA pol ε average occupancy in red [35]; (**C**) Insertion patterns of Tf1 in type II genes. Tf1 insertion in black [36], average DNA pol ε occupancy in red [37] and average Sap1 occupancy in green [38].

More mechanistic insights into the significance of these interactions came from the discovery that several bacterial transposons also show interactions between their transposition machinery and the β-clamp. The first one described was the type II element Tn7 [21]. This element has two mechanisms of insertion site selection, regulated by the choice of one of two transposon encoded specificity factors, TnsD and TnsE [22]. The first one is a highly targeted insertion mechanism dependent on sequence recognition by TnsD [23]. For its part, TnsE dependent target site selection has looser sequence requirements, but shows several particularities that suggested the involvement of replication forks. Tn7 inserts via TnsE into plasmids undergoing replicative transfer [24] with a striking bias of insertion orientation that correlates with the directionality of replication. Additionally, TnsE can guide insertion into the host chromosome favoring replication termination sites and showing the same orientation bias [25] (Figure 2A). These observations suggested that TnsE could detect the presence of replication forks and direct transposition towards them. TnsE binds to substrates with recessed 3′ ends that could occur in replication forks, providing a potential explanation [26]. The mechanism for this target site selection pathway was explained when sequence conservation analysis of TnsE revealed a consensus β-clamp interaction motif [21]. In agreement with a potential role for a TnsE/β-clamp interaction in Tn7 mobility, mutation of this motif lowered transposition activity in vivo, and β-clamp overexpression increased it. A minimal in vitro transposition system with a gapped substrate to provide the recessed 3′ end enables efficient transposition, but with random position and orientation with respect to the gap in the target. However, loading the β-clamp onto the target restored the site specificity and dramatic orientation bias of the insertions. It appears that Tn7 specifically targets discontinuous DNA replication for insertion through interaction with the β-clamp [27].

Since this work was published multiple other bacterial transposons, utilizing very different insertion mechanisms, have revealed interactions between their transposition machinery and the β-clamp. The IS200/IS605 family of transposons uses a single-stranded DNA (ssDNA) “peel and paste” transposition mechanism that is profoundly influenced by replication fork dynamics [28,29,30]. Excision of IS608 and ISDra2, belonging to this family, is more efficient when the transposed strand is in the lagging strand template, transiently providing a ready ssDNA donor after passage of the replication fork. At the insertion side of the reaction the fork also has a strong influence, because it preferentially targets, again, the lagging strand template. As a consequence, the orientation of members of this family of transposons recapitulates the directionality of DNA replication in their hosts [30]. Notably, the IS608 TPase TnpA binds β-clamp by yeast two hybrid, and also shows affinity for fork-like structures [31], suggesting that, despite the profound differences in insertion mechanisms, IS200/IS605 and Tn7-like transposons could use common targeting strategies. These mechanisms may turn out to be very common: multiple IS families exhibit interactions between their TPases and β-clamp, also showing similar orientation biases with respect to host DNA replication [32].

The interactions between transposition machinery and sliding clamps even transcend the division between type II DNA and type I RNA-intermediate transposons: a proteomic survey of the human non-LTR retrotransposon long interspersed nuclear element-1 (LINE-1) ribonucleoprotein revealed the interaction between the endonuclease (EN)/reverse transcriptase (RT) ORF2p and PCNA, carried out via a canonical PIP-box [39]. This interacting motif is necessary for transposition activity. Interaction with PCNA was decreased in ORF2 EN and RT mutants, indicating that it is recruited in the context of the initial steps of LINE-1 transposition. In contrast with IS200/IS605 and Tn7, the mechanism of LINE-1 target-primed reverse transcription insertion does not readily provide an explanation for the involvement of PCNA, but potential roles in RT processivity or post-insertion DNA repair can be imagined without the involvement of a native DNA replication fork [39].

A common observation in the study of replication regulated transposons is the insertion preference for sites of programmed fork arrest [26,30]. Such sites are an essential part of the host replication program, because they organize the genome in domains with defined replication directionalities, usually disfavoring replication in antisense orientation over highly expressed genes [40,41,42]. This organization prevents head-on collisions between the advancing replisome and transcription complexes, which can result in replisome loss, leaving unreplicated regions that become fragile sites upon chromosome segregation [43]. Programmed replication fork barriers (RFB) usually require the action of sequence-specific DNA binding factors with asymmetric binding properties that impart a defined polarity to the barrier activity, blocking fork advance in one direction but allowing progression in the opposite direction [44]. The replication fork can also arrest when it encounters other types of impediments to its progression, such as G-quadruplexes, highly transcribed genes, or tightly-bound DNA binding proteins [45]. The dynamics of arrested forks is a subject of intense research because forks that stall, losing the replisome, can destabilize leading to double-strand breaks and gross chromosomal rearrangements. Unsurprisingly, arrested forks often activate DNA damage signaling pathways and engage repair mechanisms [46].

Since bacterial replication usually starts from a single origin, with two sister forks travelling around the circular chromosome, their termination and merging sites are well known. Both Tn7 and IS200/IS608 transposons exhibit preference for natural and ectopic replication termination sites [25,30] (Figure 2A). A possible explanation invokes the role of DNA replication fork structure in the insertion mechanisms of these elements: a stalled fork would exhibit the ssDNA target for a longer period, until DNA replication from a converging fork merges with it, perhaps providing an extended window of opportunity for transposition to occur. In agreement with this potential mechanism the bacterial protein that binds and protects single-stranded template DNA during replication, Ssb, is a negative regulator of IS608 transposition [31]. Several TPases show binding to unique DNA structures that could be exposed in stalled replication forks [26,31]. Interaction between the sliding clamp and transposition machinery might also be favored in stalled replisomes, because the loss of DNA polymerases could release the hydrophobic pockets in the clamp [18]. In addition, sliding clamps are involved in the signaling of DNA damage and the recruitment of repair activities, both of which could potentially modulate interactions with the transposition machinery at sites of fork arrest.

Recent work in fungal LTR retrotransposons also point to the involvement of replication fork arrest in their insertion target site selection. The high gene density in fungal genomes puts a selective pressure on these elements to evolve strategies that target insertion away from protein coding sequences, so as not to decrease host fitness. These strategies usually take the form of protein-protein interactions between the integrase (INT) and host DNA binding factors that localize at non-coding regions such as promoters and heterochromatin, providing a platform to recruit the integration complex (intasome) to “safe haven” targets [47]. Intense scrutiny has revealed the insertion preferences of the copia-like Ty1 and Ty5 as well as the gypsy-like Ty3 elements in *Saccharomyces cerevisiae*, and the gypsy-like Tf1/2 elements in *Schizosaccharomyces pombe*. These closely related elements show a variety of preferred target sites: Ty1 and Ty3 insert upstream of type III genes (*tDNA*, *5S* and *U6*; Figure 2B), Ty5 inserts in heterochromatic domains, and Tf1/2 insert in promoters of protein-coding genes. (Figure 2C) Potential INT DNA binding partners that have been identified could explain these insertion preferences. Ty1 INT interacts with the RNA Pol III subunit AC40 [48], and substituting it with a non-interacting ortholog leads to dispersal of insertions away from its usual targets in type III promoters. Ty3 can transpose in vitro into *tDNA* targets in the presence of transcription factor for polymerase III B (TFIIIB)/transcription factor for polymerase III C (TFIIIC) [49]. Ty5 INT binds to the silencing factor Sir4 [50], and the interacting domain can be transferred to a different sequence specific binding factor that can then direct insertion to ectopic binding sites [51].

The fission yeast element Tf1 element, like its close relative Tf2, shows insertions in type II protein coding gene promoters [52,53]. While interactions between Tf1 INT and host factors have been described, none fully explain this insertion specificity. The transcription factor Atf1 binds INT [54], but deletion mutants don’t exhibit decreased transposition and only show a modest difference in target site preference. Together with the clear accumulation of insertions in the nucleosome-depleted regions (NDR) that are usually present in type II promoters, this led to a model whereby chromatin structure and sequence-specific DNA binding factors collaborated to determine Tf1/2 target site preferences [53].

The DNA binding factor Sap1, which also binds Tf1 INT by yeast two-hybrid analysis, is the main determinant of NDR formation in pombe genes [55]. Genome-wide analysis showed that Sap1 binding is highly predictive of Tf1 insertion [36,56]. However, Sap1 binding is not sufficient for insertion, as some very strong Sap1 binding sites are cold spots for transposition. Sap1 has an additional function required for genome integrity: in certain binding arrangements, it forms a polar RFB [57,58,59]. A mutation in *sap1* that abrogates this function but only mildly affects DNA binding [38] severely decreases Tf1 transposition [36]. Additionally, Sap1 binding sites constitute insertion hotspots but only if they exhibit RFB activity, and their insertion competence depends on the orientation with respect to the advancing fork. However, other programmed RFB that are independent of Sap1 are not targeted for insertion [36,53,56]. These observations indicate that Sap1 binding and RFB activity are both necessary but neither is sufficient for target site selection. Measuring intasome recruitment by chromosome conformation capture (3C) between the mature cDNA and an ectopic target site revealed that fork arrest is necessary for intasome tethering to the target. Together, these results suggest that Sap1 presence and its RFB activity collaborate to determine target site selection [36].

Unlike in the case of β-clamp interacting TPases, there is no obvious mechanism for the recognition of an arrested form by the LTR retrotransposon integrase. Could the arrested fork be the real tethering factor? Sap1-INT interaction is only detectable by yeast two-hybrid [36,56]. Weak intasome tethering can be detected at Sap1-independent RFB when in the blocking orientation with respect to fork progression, although these are not insertion targets [36,53]. The Sap1 binding and RFB requirements are separable, so a model in which the arrested fork tethers the intasome and the Sap1 interaction activates it for insertion could have merit.

Comparing the insertion preferences of fungal LTR retrotransposons may offer new insights. Type III genes, the targets for Ty1 and Ty3 insertion, are notorious RFB [60,61], and Sir4, the Ty5 tethering factor, is recruited to sites of replication fork arrest [62]. In the amoeba *Dictyostelium discoideum* several LTR and non-LTR retrotransposons also show targeting to *tDNA* genes [63]. These target site preferences could indicate an ancestral role of arrested replication forks in retrotransposon target site selection. The Tf1-like element Tj1 originating from the fission yeast *Schizosaccharomyces japonicus* can be coaxed into transposing in *S. pombe* [64]. Tj1 is present in heterochromatic regions of the *S. japonicus* centromeres, which exhibit dense clusters of *tDNA* [65]. Unlike its close cousin Tf1, the Tj1 insertion points in *S. pombe* accumulate in a small window upstream of type III genes, reminiscent of the insertion pattern observed in Ty3. In conclusion, the insertion site preference for type III and type II promoters and heterochromatin appear to be characteristic of fungal LTR retrotransposons, but the choice of one of these targets is not tied to particular families of elements, with members of Ty1/Copia, Ty3/Gypsy and Tf1/Gypsy groups showing insertion preferences as variant as all fungal LTR taken as a whole. The only commonality in all these target types is their activity as RFB.

Why are transposons fixated on the replication fork? Insertion into fork arrest sites does not impart an obvious selective advantage to the mobile element. A potential role could be to widen the potential host spectrum, increasing their chances for horizontal transfer (HT). HT is essential for the evolutionary success of transposons, because it allows them to escape vertical extinction. Despite its importance, HT is extremely poorly understood.

Since transposons rely on the cellular machinery for their vertical transmission, they may evolve specialized adaptations to the new host that ensure their persistence by increasing their copy number to avoid loss by genetic drift. Conversely, the host evolves with its transposons, defending against their destabilizing influence, and sometimes domesticating the transposon machinery, exapting it into new cellular activities [66]. This tug-of-war between the host and the transposon guides their co-evolution [67]. However, these host-specific TE adaptations may not serve after HT to a new host, and could even be detrimental. Most HT events described in eukaryotes occurred between closely related species [68], perhaps as a consequence of host specialization, but the ubiquity of some families of transposons indicates that wide leaps, even between different phyla, do occur in nature. The evolutionary success of a transposon could therefore depend not only on host-specific adaptation to ensure vertical transmission, but also on balancing generalist mechanisms that enable successful HT.

The replication fork is one potential focus point for these generalist interactions. The structure of the replicating DNA is completely universal in all cellular life forms. A transposon able to exploit this structure to facilitate its transposition would always find the same substrate no matter the host [27]. The protein factors that carry out DNA replication are also remarkably conserved, because the essential nature of this process subjects them to intense purifying selection. Interaction between sliding clamps and replication factors constitutes another universal feature DNA replication, providing transposons with a conserved point of cross-talk with the fork [21,69,70]. Convergent evolution of these interactions may explain the widespread presence of β-clamp and PCNA binding motifs in transposition machinery. A central role of sliding clamp interactions in HT was recently proposed, with supporting mechanistic evidence, in the IS1634 element from the bacterium *Acidiphilum* sp. [70]. Mutating a β-clamp binding motif present in its TPase showed that transposition efficiency is directly proportional to binding affinity, not only in its *Acidiphilum* host but also upon transfer to *E. coli*. This work also showed that *Acidiphilum* IS1634 TPase can interact with the archaeal PCNA sliding clamp in *Methanosarcina*. Conversely, an IS1634 element TPase aboriginal to *Methanosarcina* can interact with the *Acidiphilum* β-clamp. These experiments dramatically illustrate the generalist nature of interactions between sliding clamps and transposition machinery, and suggest that the similitude between the β-clamp interaction motif and the PIP-box might enable transposon HT between host species belonging to entirely different kingdoms.

The search for insertion safe havens may also benefit from an ancestral preference for arrested replication forks. Since they coordinate the direction of replication and transcription they are usually localized in intergenic regions, making them an attractive platform for new mobilizations minimizing the mutagenic potential. RFB stop fork progression through poorly understood mechanisms but they are usually associated with tight protein-DNA interactions [44,71]. Several elements that show RFB activity, such as promoters bound by transcription factors and highly compacted heterochromatin [60,62,72], would constitute safe havens in a broad variety of potential hosts. Here again, experimentally forced horizontal transfer could provide interesting information about what insertion targets are available to a transposon undergoing HT [64].

## 4. Influence of TEs Presence in Host DNA Replication and Homologous Recombination

Ever since their discovery, TE were observed to very strongly destabilize their surroundings. Mutations created by transposition into cellular genes or regulatory elements show high rates not just of reversion (often caused by TE excision) but also of derivation into different alleles affecting the same gene [73]. Moreover, TE can cause gross chromosomal rearrangements involving their insertion sites [74]. In the case of type II DNA TE this phenomenon is often explained by the activity of the transposition machinery, which can lead to erroneous excisions involving dispersed TE sequences. Due to the ease of generation of derived alleles, much of the early research into TE after their re-discovery in bacteria, fungi and animals concentrated in the characterization of these post-insertion rearrangements, leading to pilot models of transposition mechanisms [3].

But mobilization is not the only cause of TE-mediated rearrangements. *S. cerevisiae* mutations caused by LTR retrotransposon insertion also exhibit instability [75,76]. However, since the INT protein binds to the free cDNA ends, not the integrated element, the transposition mechanism can’t explain the rearrangements. Instead, they depend on the host Homologous Recombination (HR) pathway. TE mediated rearrangements showing the hallmarks of HR are common in all organisms. Repetitive DNA is intrinsically unstable because the process of HR includes a search for homology that in repeated sequences may engage non-allelic loci, resulting in cross-over and non cross-over resolution, observable as rearrangements and gene conversions. As a result, HR of TE sequences was considered an inevitable consequence of its repetitive nature. Since the only requirement for this process is sequence homology, it also involves inactive copies, which vastly outnumber active ones.

Mobilization-dependent and HR-dependent rearrangements are now known to be major drivers of eukaryotic genome structural variation (SV) and evolution [77,78,79]. Examples of structural variation involving TE, with and without adaptive value to the host, are abundant in the literature. The role of fungal LTR retroelements in yeast SV has been extensively investigated, because the small genome and long history of strain domestication facilitates comparative analysis [80]. The non-autonomous type I *Alu* elements seem to be a major cause of SV, both in polymorphisms present in human populations [81] and in primate evolution [82]. Plant genomes with high transposon content exhibit extreme SV, some of which underlies important traits in commercial cultivars [83]. Finally, TE mediated rearrangements could explain some of the genomic instability observable in cancer [84], which often shows activation of TE as part of its disregulated transcriptional program [85].

The processes that lead to mobilization-dependent SV can be retraced because transposition mechanisms are relatively well understood, sometimes revealing behaviors nothing short of acrobatic [86]. But since the role of TE in HR-mediated rearrangements was considered to be passive, it has received little attention. Work in fungal LTR retrotransposons has revealed that their behavior in this process is more active than previously thought.

The recombinogenic activity of *S. cerevisiae* LTR elements was observed even before they were recognized as TE sequences. Rothstein characterized deletion and inversion mutations of the *tDNA* gene *SUP4*, locating the breakpoint regions in five Ty1 LTR (then known as delta sequences) that flanked the locus [87]. This phenomenon required the HR factor RAD52. Soon thereafter, the characterization of revertants of mutations caused by Ty1 insertions revealed that it was frequently excised through HR between the two flanking LTR [1,75,76]. This recombination explains the abundance of solo LTR that pepper eukaryotic genomes: each represents an ancient insertion that was lost through recombination, leaving a solo LTR at the insertion site. Inter-LTR recombination is therefore a very common event. In fact, it appears to be the only process that counteracts the plant genome gigantism caused by runaway LTR retrotransposon activity [88]. HR between non-allelic LTR underlies a large proportion of yeast SV [80]. The solo LTR is sufficient to mediate HR rearrangements [89], so the destabilizing influence of LTR retrotransposons could continue even after their complete extinction from the host genome.

**Figure 3:**
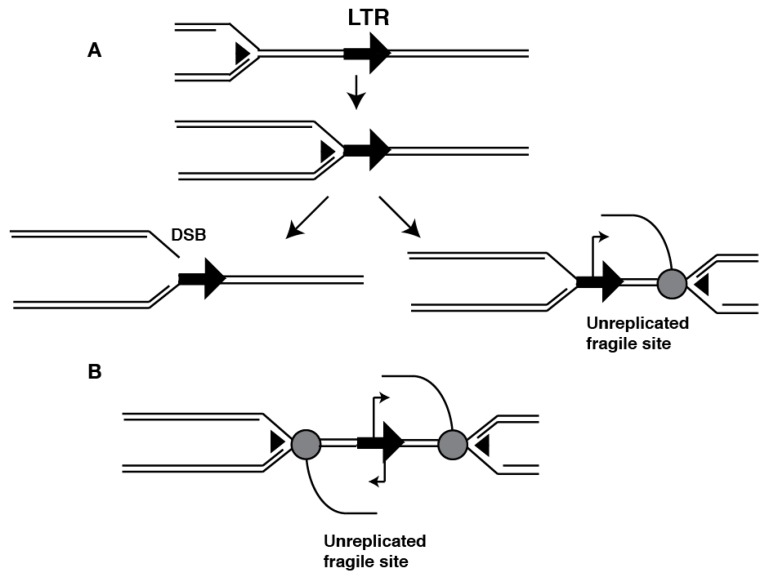
Fork instability at transposable elements (TE). An LTR containing replication fork barriers (RFB) can lead to replication fork stalling and double strand break (DSB) formation (left). (**A**) Active transcription of the TE can cause replisome-RNA Pol II collisions and unreplicated regions (right); (**B**) TE with actively transcribing bidirectional promoters can cause replisome-RNA Pol II collisions and unreplicated regions.

Paradoxically, the frequency of mitotic and meiotic non-allelic HR of Ty1 sequences is low when directly compared with artificially introduced non TE repeats [89]. Some LTR are more recombinogenic than others, even in very similar contexts, suggesting that factors extrinsic to their sequence homology or repetitive nature influence this activity, and that mechanisms that prevent TE dependent HR exist. Mutation of the topoisomerase TOP3 increases the frequency of *SUP4* deletion by inter-LTR recombination [90]. TOP3 restarts stalled replication forks together with the RecQ DNA helicase slow growth suppressor 1 (SGS1). Arrested forks engage the HR machinery to restart the replisome, and mutations in TOP3 or SGS1 result in increased HR and gross chromosomal rearrangements [91]. The dependence on TOP3/SGS1 to prevent HR of LTR indicates that these elements constitute impediments to the progression of the replication fork (Figure 3A). In agreement with this model, Ty LTR exhibit accumulation of DNA polymerase ε indicative of replisome pausing, as well as DNA damage signaling by local accumulation of phosphorylated histone γ-H2A. These hallmarks of replication fork arrest are exacerbated in mutants of RRM3, a DNA helicase that aids the replication fork in overcoming obstacles to its progression [35].

Despite their evolutionary distance with Ty elements, the *S. pombe* Tf1/2 LTR retrotransposons also exhibit this property. A genomic survey of γ-H2A localization revealed that Tf2 and solo LTR elements signaled DNA damage even during a completely undisturbed S phase [92]. Strikingly, the Tf1/2 LTR contain a conserved binding site for Sap1 (yes, the very same DNA binding factor implicated in target site selection) that exhibits polar RFB activity [38]. Sap1 is not conserved in *S. cerevisiae*, so whatever RFB activity Ty LTR have must be carried out by other mechanisms; this property could be the result of convergent evolution co-opting host factors.

Most HR at Ty elements does not occur between non-allelic copies, but instead involves gene conversion of the inserted copies by cDNA or cDNA intermediates [93,94]. A sizable proportion of mobilization events in fungal LTR retrotransposons is INT-independent, but requires HR machinery. In the case of *S. pombe* Tf2 this pathway constitutes the majority (~70%) of mobilization events observed upon overexpression of the transposon [95]. Screens for regulators of Ty mobility seldom distinguish between mobilization by insertion and HR mediated gene conversion events, so some negative regulators of mobilization could be in fact repressors of Ty mediated HR. As an example, the PCNA unloader ELG1 was independently identified as a negative regulator both of inter-LTR recombination [96] and of Ty1 mobility [97], and multiple host factors that repress mobility have known functions to repress HR. However, it is difficult to separate the contribution of LTR-initiated HR from the effect of DNA damage prevention, checkpoint, signaling and repair pathways on Ty cDNA formation and mobility. For example, mutation of SGS1 or RRM3 increases Ty1 mobility dependent on RAD52, but rather than stimulating cDNA mediated gene conversion the increase is due to the formation of cDNA multimers [98,99], which are the main mediators of mobility when INT activity is prevented [100]. The dissection of this phenomenon will require specifically designed models that address these multiple pathways.

Transcription also plays an important role in this process that is independent from cDNA generation. Inducing the transcription of a Ty1 copy via a regulated promoter increases its competence as a recipient of cDNA mediated gene conversion by up to an order of magnitude [101]. Similarly, mutations that activate transcription of Tf1/2 in *S. pombe* increase mobility by HR [102]. Tf1/2 elements are silenced by three partially redundant domesticated Pogo/Tigger TPase-like factors collectively known as centromere protein B (CENP-B). Besides Tf1/2 increased transcription, mutations in these factors also cause a dramatic loss of genome integrity and recruitment of HR factors to LTR [38]. Mutations of Sap1 abrogating RFB activity suppress the loss of genome integrity, indicating that forks arrested by Sap1 at LTR become destabilized in CENP-B mutants. As a result, CENP-B mutants require an intact HR pathway for viability. While INT-mediated mobility is not affected, HR-dependent mobility increases dramatically in a CENP-B mutant [102]. Conversely, mutation of *sap1* removing RFB activity practically eliminates INT-independent transposition by HR [36]. These observations suggest that fork arrest and transcription at the recipient elements have a synergistic effect on HR-dependent mobility.

Transcription poses a formidable obstacle to replication fork progression [43]. The presence of programmed RFB at LTR could exacerbate replication-transcription conflicts (Figure 3), leading to the genome-wide proliferation of arrested forks and unreplicated regions that engage HR to resume replication and prevent instability. Increased HR, if directed at the offending repetitive elements, could cause gross chromosomal rearrangements. This model would explain the role of heterochromatin in maintenance of genome integrity [103]. Loss of silencing of TE and other forms of repetitive elements leads to widespread replication-transcription conflicts that cause DNA damage localized at heterochromatic DNA, and rearrangements through non-allelic or improperly resolved HR. Since this source of genome instability does not require transposition mechanisms, non-autonomous and even highly mutated copies of TE could participate. This phenomenon has been observed in multiple model organisms, affecting centromeric and rDNA repeats as well as TE [38,72,104,105,106,107].

What function could RFB activity bring to these elements? A possible explanation invokes HR-mediated mobilization. An element able to exploit this process may paradoxically stabilize its presence in the host genome, perhaps counteracting inter-LTR recombination [38]. Such a mechanism would enable a transposon colony to use the cDNA pool as a community resource and a communication tool, enforcing sequence consensus or spreading variants with favorable characteristics [108,109]. Alternatively, if the RFB contained in the LTR mediate target site selection they could aid genome colonization by dispersing insertion hotspots to new safe havens. The LTR of Ty1 and Tf1/2 elements show this activity [36,110], and if extensible to other elements it could explain the tendency of transposons to accumulate as clusters and nested insertions.

Regardless of its role in TE biology, the consequences for the host genome can be quite dramatic. The proliferation of RFB could change the replication program and increase genome plasticity, particularly under conditions of active TE transcription. Since many TE are transcriptionally activated by cellular stress, TE-mediated HR could represent an additional layer of the long-proposed role of transposons in host adaptability. Gene amplification is a common mechanism for adaptation to stress. Some TE, such as the Tf1 element, show a preference for insertion in promoters of stress-regulated genes, and could therefore poise them for amplification by HR. This activity has been observed in a case of Histone gene amplification mediated by Ty1 [111] which can be induced by treatment with hydroxyurea, a drug that stalls replication, and by mutation of factors required for fork progression [112]. Similarly, experimental evolution of yeast grown under limiting glucose yields adaptive rearrangements, such as amplification of hexose transporters, through non-allelic HR between transposon sequences [113].

It is not known whether other TE present RFB like fungal LTR elements. Inverted repeats of the primate short interspersed nuclear element (SINE) *Alu* form hairpins that arrest replication forks in bacteria, yeast and mammalian cells [114]. *Alu* elements constitute the majority of inverted repeats in the human genome and could therefore influence genome plasticity via their interaction with replication and HR. The non-LTR retrotransposon LINE-1 are the most abundant autonomous TE in humans, and their role in cancer progression is the subject of much debate because multiple cancers exhibit LINE-1 transcription activation and mobilization. LINE-1 contain bidirectional promoters that, if activated, could arrest replication forks converging on the transcribed element resulting in fragile sites (Figure 3B). Oncogenic transformation is often accompanied by increased endogenous replication stress and DNA damage [84], and the resulting genomic instability that drives cancer progression could have a TE component. Increased activity of the LINE-1 transposition machinery is a likely culprit [115,116], but considering the high TE content of the human genome, and the genome integrity phenotypes of heterochromatin mutations observed in model organisms, loss of seamless repetitive element replication might also be a significant contributor [103].
